# Management of Polypharmacy and Potential Drug–Drug Interactions in Patients with Pulmonary Aspergillosis: A 2-Year Study of a Multidisciplinary Outpatient Clinic

**DOI:** 10.3390/jof10020107

**Published:** 2024-01-26

**Authors:** Dario Cattaneo, Alessandro Torre, Marco Schiuma, Aurora Civati, Giacomo Casalini, Andrea Gori, Spinello Antinori, Cristina Gervasoni

**Affiliations:** 1 Department of Infectious Diseases, ASST Fatebenefratelli-Sacco University Hospital, 20157 Milan, Italy; dario.cattaneo@asst-fbf-sacco.it (D.C.); spinello.antinori@unimi.it (S.A.); 2 Gestione Ambulatoriale Politerapie (GAP) Outpatient Clinic, ASST Fatebenefratelli-Sacco University Hospital, 20154 Milan, Italy

**Keywords:** fungal infections, pulmonary aspergillosis, polypharmacy, drug–drug interactions, outpatient clinic

## Abstract

Pulmonary aspergillosis mainly affects elderly patients, patients with pulmonary complications, patients with hematological malignancies, organ transplant recipients, or critically ill patients. Co-morbidities may result in a high rate of polypharmacy and a high risk of potential drug–drug interaction (pDDI)-related antifungal azoles, which are perpetrators of several pharmacokinetic- and pharmacodynamic-driven pDDIs. Here, we report the results of the first 2-year study of an outpatient clinic focusing on the management of therapies in patients with pulmonary aspergillosis. All patients who underwent an outpatient visit from May 2021 to May 2023 were included in this retrospective analysis. A total of 34 patients who were given an azole as an antifungal treatment (53% voriconazole, 41% isavuconazole, and 6% itraconazole) were included. Overall, 172 pDDIs were identified and classified as red- (8%), orange- (74%), or yellow-flag (18%) combinations. We suggested handling polypharmacy in those patients using specific diagnostic and pharmacologic interventions. As expected, red-flag pDDIs involved mainly voriconazole as a perpetrator (71%). However, nearly 30% of red-flag pDDIs were not related to antifungal therapy. These findings highlight the importance of conducting an overall assessment of the pharmacologic burden and the key role played by a multidisciplinary team for the optimization of therapies in patients with pulmonary aspergillosis.

## 1. Introduction

Pulmonary aspergillosis is a disease caused by a fungal infection that has gained renewed interest in the past few years as a serious complication of COVID-19 [1,2,3]. Besides coronavirus disease, invasive pulmonary aspergillosis can be seen among immunocompromised, neutropenic patients whose underlying risk factors are hematological malignancies, hematopoietic stem cell transplantation, solid organ transplant, and prolonged chemotherapy/steroid use. Invasive pulmonary aspergillosis can also be observed in non-neutropenic patients with prolonged intensive care unit stay, mechanical ventilation, previous viral pneumonia such as influenza, and chronic obstructive pulmonary disease (COPD). Otherwise, chronic pulmonary aspergillosis is observed in immunocompetent individuals with pre-existing pulmonary cavities resulting from tuberculosis, bronchiectasis, sarcoidosis, or cavitary neoplasia. Additionally, allergic bronchopulmonary aspergillosis is seen among patients with underlying cystic fibrosis or asthma [4,5,6,7,8,9,10,11,12]. This disease can also affect elderly individuals with prior or concurrent pulmonary conditions or with increased disease susceptibility (i.e., with pathophysiological lung alterations) [4,5,6]. In these patients, who are often also elderly, the presence of associated comorbidities may result in a high rate of polypharmacy and a high risk of potential drug–drug interactions (pDDIs), defined as pharmacological or clinical responses to the administration of a drug combination which is different from the known effects of the two agents when given alone.

Polypharmacy and pDDIs are two conditions that can complicate the treatment of pulmonary aspergillosis. Indeed, antifungal azoles, which are among the first-line treatment for this disease, are strong inhibitors of cytochrome P450 isoenzymes, resulting in many clinically relevant pharmacokinetic (PK)-driven pDDIs, especially when co-administered with narrow therapeutic index drugs; such immunosuppressants and/or targeted therapies are used for the treatment of hematological malignancies [12]. Additionally, these drugs could also cause some important pharmacodynamic (PD) interactions, such as potential additional effects of voriconazole if combined with other drugs known to cause QT prolongation [12,13,14].

For these reasons, in May 2021, we expanded our outpatient clinic for the management of polypharmacy to include patients with pulmonary aspergillosis (Gestione Ambulatoriale Politerapie [GAP]-Fungi) using the same methodology previously described in people with HIV (GAP) and in patients with mycobacterial infections (GAP-MyTB) [15,16]. The main aims of the GAP-Fungi clinic are to assess whether the patients are treated with drug combinations which are contraindicated because of known or predictable DDIs, evaluate the clinical relevance of the pDDIs, and provide written advice as to how the treatments should be modified where possible. We describe the results of the first 2 years.

## 2. Materials and Methods

### 2.1. Patient Selection and Study Design

Demographic characteristics, localization and type of aspergillus infections, antifungal therapy, and number/class of co-medications from patients included in the GAP-Fungi database from May 2021 to May 2023 were collected. For the diagnosis of invasive aspergillosis in immunocompetent or immunocompromised patients, the “Criteria of Consensus definitions of the Infectious Diseases Group of the European Organization for Research and Treatment of Cancer and the Mycoses Study Group (EORTC-MSG): invasive aspergillosis in immunocompetent or immunocompromised patient” were used [17] The criteria for COPD patient with GOLD stage III or IV with recent exacerbation of dyspnea, proposed by Bulpa et al., were used for the diagnosis of pulmonary aspergillosis [18]. For chronic pulmonary aspergillosis, we used the clinical guidelines of an expert group from the European Society for Clinical Microbiology and Infectious Diseases and from the European Respiratory Society [19].

The overall risk of pDDIs between all administered drugs was assessed using INTERcheck WEB (https://intercheckweb.marionegri.it, accessed on 13 December 2023), Medscape Drug Interaction checker (https://reference.medscape.com/drug-interactionchecker, accessed on 13 December 2023), and UpToDate (https://www.uptodate.com/drug-interactions, accessed on 13 December 2023). PDDIs were classified as red-flag (drug combinations that should be avoided), orange-flag (drug combinations that may require close monitoring and/or drug dose adjustments for potentially serious clinical consequences), or yellow-flag (drug combinations with minor clinical relevance) combinations based on their severity and clinical relevance. The first scoring of the severity of each DDI was carried out independently by DC and subsequently reassessed by CG. Then, the two experts collegially discussed and scored the pDDIs while taking into account the clinical condition of each individual patient based on their experiences. A final report summarizing the risk of pDDIs and providing practical suggestions on how to change therapy (if needed) was given to the attending physicians.

There were no predefined criteria to determine the therapeutic intervention. Every case was discussed collectively and approached individually for each patient, taking into account peculiar clinical features, pharmacologic data, and the experience gained in over 10 years of managing patients with heavy polypharmacy and a high risk of DDIs.

The burden of medications with anticholinergic effects was also estimated in patients >65 years using the anticholinergic burden (ACB) score [20,21,22]. Particular attention was given to patients with an ACB score of >3, which is a value associated with a significantly increased risk of adverse events, including cognitive impairment and falls [20,21,22].

At the end of the GAP-Fungi visit, a written report was provided for the attending physicians. In this report, we summarized the pDDIs based on clinical relevance, proposed the additional diagnostic interventions [i.e., therapeutic drug monitoring (TDM) of azoles and/or co-medications], and provided suggestions on how the pharmacologic treatments should be eventually modified. This study was approved by our Ethics Committee [Comitato Etico Interaziendale Area 1, Milan, Italy (Protocol No. 11903)]. All patients signed a written informed consent form for medical procedures/interventions performed for routine treatment purposes, according to the Ethics Committee (Comitato Etico Interaziendale Area 1, Milan, Italy).

### 2.2. TDM of Antifungal Azoles

Plasma trough concentrations of voriconazole, isavuconazole, and itraconazole were measured using a liquid chromatography method coupled with tandem mass spectrometry. Briefly, 50 μL of plasma was purified through protein precipitation with a solution of methanol and acetonitrile, and after dilution with water, plasma was injected. Chromatographic separation was obtained with a C18 XBridge column (Waters, Milan, Italy) under gradient conditions with a mobile phase of CH3COONH4 2 mmol/L 0.1% formic acid (solvent A) and 0.1% formic acid in methanol (solvent B). All azoles were quantified using a multiple-reaction monitoring mode. The method was linear from 0.2 mg/L to 20 mg/L for isavuconazole and voriconazole and from 0.2 to 10 mg/L for itraconazole. The performance of the method was tested during each analytical run using internal quality controls (only analytical runs with imprecision and inaccuracy of <15% were accepted). The following therapeutic ranges were considered for the three azoles: voriconazole range of 1.5–5.0 mg/L, isavuconazole range of 1.0–5.0 mg/L, and itraconazole range of 0.5–1.5 mg/L [23].

### 2.3. Statistical Analyses

Continuous variables were given as mean values ± standard deviation. Categorical data were given as absolute numbers and percentages. Differences in the frequency distribution between groups were assessed using Pearson’s chi-squared test.

## 3. Results

### 3.1. Patient Characteristics and Antifungal Treatment

A total of 34 patients were included in the GAP-Fungi database (Table 1). They were mostly men (56%) and Caucasian (82%) with a mean age of 67 ± 12 years (62% were over 65 years old). The fungal infections were mainly caused by Aspergillus fumigatus (12 patients) isolated from a respiratory specimen. In 44% of the cases, aspergillosis was diagnosed in the presence of (a) chronic pulmonary and/or systemic symptoms, (b) radiographic findings consistent with aspergillosis (i.e., nodules or cavities) after excluding other causes, and/or (c) positive serum or bronchoalveolar lavage galactomannan, with the detection of Aspergillus fumigatus IgG antibodies (using an enzyme-linked immunosorbent assay, Bordier Affinity Products, Crissier, Switzerland) or Aspergillus IgG (using a Western blot, LDBIO, Lyon, France) through an aspergillus polymerase chain reaction test. All patients were treated with azoles as monotherapy (53% voriconazole, 41% isavuconazole, and 6% itraconazole).

### 3.2. Co-Medications, pDDIs, and ACB Scale

**Figure 1:**
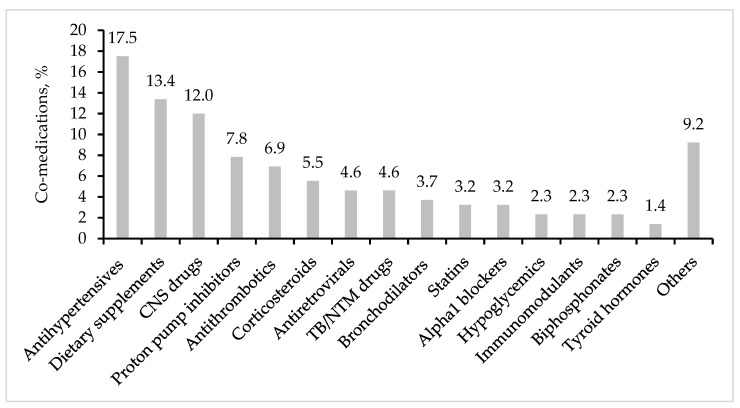
Distribution of the main drug classes of co-medications in the 34 patients with pulmonary aspergillosis included in the GAP-Fungi database (data are given as percentages of the total of non-antifungal prescriptions).

A total of 33 out of the 34 patients enrolled in the GAP-Fungi database were given 7.2 ± 3.7 concomitant medications (ranging from 1 to 16 drugs for a total of 217 prescriptions) in addition to antifungal therapy for a total of 8.2 ± 3.7 drugs (one patient only received antifungal therapy). As shown in Figure 1, the most commonly prescribed co-medication classes were antihypertensives (18%), dietary supplements (13%), central nervous system (CNS) drugs (12%), proton pump inhibitors (PPIs, 8%) and antithrombotics (7%).

In total, 172 pDDIs were identified involving equally antifungal (49%) and non-antifungal drugs (51%). No significant differences in the scoring of pDDIs were found when comparing antifungal drugs with co-medications concerning orange-flag (48% vs. 52%) or yellow-flag (43% vs. 57%) pDDIs (Table 2). Conversely, 10 and 4 out of the 14 red-flag pDDIs involved, respectively, antifungal agents and co-medications (71% vs. 29%; *p* < 0.05). As shown in Table 3, these pDDIs resulted mainly in an increased risk of QT prolongation (36%), impaired respiratory function (29%), and rhabdomyolysis or statin-related muscle toxicity (14%). The remaining pDDIs involved voriconazole and its inhibitory effects on the metabolisms of co-medications. Only one patient had an ACB score of >3 (Caucasian male, aged 75 years); the other patients had a mean ACB of 1.0 ± 1.2.

### 3.3. Proposed Actions Identified during the GAP-Fungi Visits

Proposed actions have been identified in 33 out of the 34 patients (97%) enrolled in the GAP-Fungi database. These actions were divided into diagnostic interventions and changes in pharmacological therapies (Table 4). Among the first, TDM of both antifungal agents (14) and co-medications (6, such as antidepressants, antiepileptics, and immunosuppressants) was proposed for 28% of patients. Electrocardiogram and electrolyte monitoring were suggested for 24% and 13% of patients, respectively.

Among the pharmacologic interventions, the most frequent action was to stop (or reduce) the PPI (33%), followed by the suggestion to stop using inhaled corticosteroids (18%) and changes in the statin (16%, reduction in the dose or change with another statin with a lower risk of pDDI). In 11% of cases, we proposed changing the alpha 1-adrenoceptor blocker (i.e., from tamsulosin to doxazosin) to limit/prevent the development of excessive hypotension, vomiting, and diarrhea related to the inhibitory effects of voriconazole on the metabolism of these drugs. All of the recommendations were applied by the clinicians running the outpatient clinic to which patients with fungal infections are referred in our hospital.

### 3.4. TDM of Azoles

Concomitantly with the GAP-Fungi visit, the TDM was available for 31 out of the 34 patients (91%). Sub-therapeutic drug trough concentrations were found in 19% and 15% of treated patients, respectively, with voriconazole and isavuconazole; only one patient given voriconazole and two patients given isavuconazole had drug trough concentrations of > 5 mg/L. Seventy-one percent of patients had drug concentrations falling within the therapeutic ranges (Table 5).

## 4. Discussion

Here, we describe the 2-year experience in GAP-Fungi, an outpatient clinic aimed at optimizing therapies in patients with pulmonary aspergillosis, as previously reported in the field of HIV and mycobacterial infections [15,16]. A high rate of polypharmacy was also observed in this population, with a mean of seven drugs concomitantly prescribed in addition to antifungal monotherapy; interestingly, only one patient referred to the outpatient service for the treatment of fungal infections in our hospital did not take co-medications. The heavy polypharmacy resulted in a high rate of overall pDDIs, which were equally distributed between antifungal therapy and co-medications. However, when analyzing the results more deeply, we found that the large majority (around 70%) of red-flag DDIs were mainly driven by voriconazole and involved its potential to (a) increase the toxicity of co-medication by inhibiting drug metabolism (PK-driven DDI) and (b) exert additive effects on QT prolongation with an increased risk of cardiotoxicity (PD-driven DDI) [12,13,14,20]. Nearly 30% of the red-flag pDDIs involved an increased risk of respiratory dysfunction, a condition mainly ascribed to a combination of non-antifungal agents (i.e., isoniazid with fentanyl, peridopril with pregabalin, and propranolol with umeclidinium/vilanterol). If such combinations cannot be avoided, we need to try our best to limit their potential adverse events by strictly monitoring cardiac and respiratory function, as well as optimizing voriconazole and co-medication exposure by TDM, as suggested in our reports for the attending physicians.

Among the pharmacological interventions, we proposed more frequently to stop/reduce PPIs. Remarkably, 50% of patients from GAP-Fungi were in fact undergoing chronic treatment with PPIs. These data, in agreement with previous findings in COVID-19 patients and in those with mycobacterial infections, confirm the chronic overuse of PPIs in different real-life settings, and the underestimation of their potential for causing clinically relevant DDIs [15,24,25,26,27,28,29]. Taken together, this evidence calls for actions to promote the correct use of PPIs, ideally limiting their prescriptions.

Thirty-five percent of our patients were undergoing treatment with inhaled corticosteroids. This is not surprising, considering that 12 out of the 34 patients had COPD. The use of these drugs in the context of pulmonary aspergillosis is a matter of debate for the need to balance the potential beneficial and harmful effects. Indeed, Kosmidis et al. recently reported that inhaled steroids in patients with chronic pulmonary aspergillosis were associated with lower mortality mainly due to a reduction in inflammation [30]. On the other hand, extensive evidence is also available showing that the isolation and colonization of *Aspergillus* spp. in immunocompromised patients (i.e., patients with cystic fibrosis and transplanted patients) are directly associated with the duration of inhaled corticosteroid treatment [31,32,33]. After a careful assessment, we decided to stop administering inhaled corticosteroids to 60% of the treated patients, whereas in 40% of cases, corticosteroids were maintained.

Unexpectedly, only 1 out of the 34 patients from the GAP-Fungi clinics had an ACB score of > 3. This differs from the recent findings in the GAP-MyTB clinics, in which we found an ACB of > 3 in nearly 20% of patients [15]. These data could suggest an increased awareness of physicians of the risks associated with the cumulative effect of anti-cholinergic drugs or, alternatively, the presence of differences in the demographic/clinical features between two cohorts of patients. Indeed, in the GAP-MyTB, we had fewer Caucasians and more patients with HIV co-infection and a history of intravenous drug use, and all conditions were often associated with drugs acting on the CNS, eventually resulting in a high ACB score. 

The GAP-Fungi clinic is an on-demand service requested by colleagues in the presence of heavy polypharmacy or a high risk of pDDIs. Currently, we see patients when antifungal therapy has already been optimized for clinical practice; most of our patients were treated with voriconazole, whereas the remaining patients were shifted to other azoles mainly for contraindicated DDIs, the inability to maintain voriconazole trough concentrations within the therapeutic ranges, or the development of severe drug-related adverse events. We believe, however, that the GAP service should be ideally performed at the bedside, when the patients are still in the hospital or in the outpatient clinic before starting treatment.

This would allow for a more accurate assessment of the pDDIs and the selection of tailored antifungal therapies associated with better treatment tolerability and optimized drug dosing.

At the time of the GAP-Fungi visit, more than 90% of the patients concomitantly had a request for the TDM of azole trough concentrations. The large majority of trough azole concentrations were in the therapeutic range; this was the result of a fine modulation of the drug dosing based on previous TDM assessments, as documented by the heterogeneity of daily doses of voriconazole. TDM is considered a mandatory tool to optimize voriconazole and itraconazole therapy; the utility of the TDM of isavuconazole is presently less defined, but it can provide very useful information in complex patients [23].

Previous studies have dealt with the issue of pDDIs in patients with pulmonary aspergillosis treated with antifungal azoles [34,35,36,37]. The novelty of our study relies on the thorough assessment of the pharmacologic burden of our patients, focusing not only on the assessment of pDDIs between antifungal and non-antifungal drugs, but also assessing the pDDIs between non-antifungal drugs (regardless of azoles). Worthy of mention, we found that nearly 30% of red-flag pDDIs were not related to azoles but involved medications prescribed by specialists other than the infectious disease physicians that were already present before starting the antifungal therapies. We believe that those findings highlight the importance of an overall assessment of the pharmacologic burden before starting antifungal therapy. The systematical description of the diagnostic and pharmacologic interventions provided to the physicians involved in the management of patients with pulmonary aspergillosis represents, in our mind, an additional strength of the present investigation. 

A potential limitation of the present investigation is represented by the retrospective design, which may have introduced bias and confounding factors. For instance, PPIs only accounted for 8% of the co-medications administered, yet they were the source of 33% of the pharmacologic interventions, suggesting a potential overestimation of the pharmacologic interventions. Moreover, it must be underlined that this is a single-center experience based on a small sample size with two conditions, which may limit the generalizability of the findings. Nevertheless, we are confident that our study could be instrumental in underlining the importance of a multidisciplinary approach to recognize the high prevalence of polypharmacy and the risk of pDDIs in patients with pulmonary aspergillosis.

In conclusion, a high prevalence of polypharmacy and red-flag pDDIs involving antifungal and non-antifungal therapies was observed in patients with pulmonary aspergillosis. A multidisciplinary team could play a key role in the optimization of pharmacological therapies in those patients.

## Figures and Tables

**Table 1:** Clinical features of the patients included in the database of the GAP-Fungi clinic.

Characteristics	Data
Patients, n	34 (56% males)
Mean age, years	67 ± 12
Ethnicity, %	Caucasian, 82% Latin, 12% Other, 4%
Pathogen, n	*A. fumigatus*, 11 *A. niger*, 6 *A. flavus*, 1 *A. fumigatus e niger*, 1
Type of infection, n	Chronic pulmonary aspergillosis, 24 Pulmonary aspergillosis in COPD, 8 - proven, 1 - probable, 7 Invasive pulmonary aspergillosis, 2 - proven, 1 - probable, 1
Localization, n	Lung, 32 Trachea and lung, 1 Sphenoid sinus and lung, 1
Antifungal treatments, n (%)	Voriconazole, 18 (53%) - 200 mg twice daily, 11 - 300 mg twice daily, 3 - 200 + 100 mg, 2 - 100 mg twice daily, 1 - 50 mg twice daily, 1 Isavuconazole, 14 (41%) - 200 mg once daily, 13 - 100 mg once daily, 1 Itraconazole, 2 (6%) - 100 mg twice daily - 300 mg twice daily
Comorbidities, n	Hypertension, 16 TB/NTM, 12 (concomitant, 3) COPD, 12 Neoplasms, 10 (concomitant, 3) Diabetes mellitus, 7 COVID-19, 6 Myocardial infarction, 4 HIV, 4

TB: tuberculosis; NTM: non-tuberculous mycobacteria; COPD: chronic obstructive pulmonary disease.

**Table 2:** Total number of drugs, potential drug–drug interactions (pDDIs), and anticholinergic burden recorded in the database of the GAP-Fungi clinic.

Type of pDDIs	Overall	Antifungal-Drugs	Co-Medications
Drugs, n	8.2 ± 3.7	1.0 ± 0.0	7.2 ± 3.7
pDDIs, n (%)	172	84 (49%)	88 (51%)
Red-flag pDDIs, n (%)	14	10 (71%) *	4 (29%)
Orange-flag pDDIs, n (%)	128	61 (48%)	67 (52%)
Yellow-flag pDDIs, n (%)	30	13 (43%)	17 (57%)
ACB ≥ 3, n	1		

pDDI: potential drug–drug interactions; ACB: anticholinergic cognitive burden scale; * *p* < 0.05 vs. co-medications.

**Table 3:** Red-flag pDDIs recorded in the database of the GAP-Fungi clinic.

Red-Flag pDDI	Potential Adverse Event
Voriconazole + alfuzosin	Both drugs increase QTc interval
Voriconazole + atorvastatin	Voriconazole increases the level or effect of the statin (risk of rhabdomyolysis)
Voriconazole + budesonide	Voriconazole increases the level or effect of budesonide by affecting drug metabolism
Voriconazole + fentanyl	Voriconazole increases the level or effect of fentanyl (risk of respiratory depression)
Voriconazole + hydroxychloroquine	Both drugs increase QTc interval
Voriconazole + indapamide	Both drugs increase QTc interval
Voriconazole + mirtazapine	Both drugs increase QTc interval
Voriconazole + simvastatin	Voriconazole increases the level or effect of the statin (risk of rhabdomyolysis)
Voriconazole + venetoclax	Voriconazole increases the level or effect of venetoclax by affecting drug metabolism
Hydroxychloroquine + formoterol	Both drugs increase QTc interval
Isoniazid + fentanyl	Isoniazid increases the level or effect of fentanyl (risk of respiratory depression)
Peridopril + pregabalin	Coadministration results in additive risk of angioedema and respiratory compromise
Propranolol + umeclidinium/vilanterol	Beta-blocker diminishes the bronchodilatory effect of beta 2-agonist

**Table 4:** Interventions suggested at the end of the GAP-Fungi visits (in addition to routine care).

Diagnostic Intervention	Frequency, n
Perform therapeutic drug monitoring	20 (28%)
Perform electrocardiogram	17 (24%)
Monitor serum electrolytes	9 (13%)
Monitor blood pressure	8 (11%)
Monitor liver function and CPK	8 (11%)
Monitor metabolic assessment	3 (4%)
Monitor renal function	3 (4%)
Monitor respiratory functionality	3 (4%)
Monitor thyroid hormones	1 (1%)
Changes in pharmacologic therapies	Frequency, n
Reduce/stop proton pump inhibitor	15 (33%)
Stop inhaled corticosteroid	8 (18%)
Reduce/change statin	7 (16%)
Change alpha 1-adrenoceptor blocker	5 (11%)
Reduce/stop benzodiazepine	3 (7%)
Reduce/change oral anticoagulant	2 (4%)
Change antihypertensive (perindopril/indapamide)	1 (2%)
Stop antidepressant (mirtazapine)	1 (2%)
Stop analgesic (fentanyl)	1 (2%)
Reduce chemotherapy (venetoclax)	1 (2%)
Reduce anticholinergic burden	1 (2%)
Patients with no suggestions	1 (2.9%)

CPK: creatinine-phosphokinases.

**Table 5:** Summary of therapeutic drug monitoring results of antifungal azoles.

Azole	TDM/Patients n/n	Mean ± SD (min–max)	Samples below the Target, n (%)	Sample above the Target, n (%)
Isavuconazole	13/14	3.0 ± 1.7 (0.5–6.5)	2 (15%)	2 (15%)
Itraconazole	2/2	1.1; 1.4	-	-
voriconazole	16/18	3.3 ± 2.0 (0.6–9.0)	3 (19%)	2 (13%)

SD: standard deviation; min: minimum; max: maximum.

## Data Availability

Data are contained within the article.
